# IoT in the Wake of COVID-19: A Survey on Contributions, Challenges and Evolution

**DOI:** 10.1109/ACCESS.2020.3030090

**Published:** 2020-10-12

**Authors:** Musa Ndiaye, Stephen S. Oyewobi, Adnan M. Abu-Mahfouz, Gerhard P. Hancke, Anish M. Kurien, Karim Djouani

**Affiliations:** 1 Department of Electrical EngineeringCopperbelt University 108291 Kitwe 10101 Zambia; 2 French South African Institute of Technology (FSATI), Tshwane University of Technology 56412 Pretoria 0001 South Africa; 3 College of Automation and Artificial IntelligenceNanjing University of Posts and Telecommunications 12577 Nanjing 210023 China; 4 Council for Scientific and Industrial Research Pretoria 0083 South Africa; 5 Department of ElectricalElectronic and Computer EngineeringUniversity of Pretoria 56410 Pretoria 0028 South Africa; 6 LISSI LaboratoryUniversity Paris-Est Creteil (UPEC) 94000 Creteil France

**Keywords:** Artificial intelligence, big data, COVID-19, data sharing, internet of things, pandemic management

## Abstract

The novel coronavirus (COVID-19), declared by the World Health Organization (WHO) as a global pandemic, has brought with it changes to the general way of life. Major sectors of the world industry and economy have been affected and the Internet of Things (IoT) management and framework is no exception in this regard. This article provides an up to date survey on how a global pandemic such as COVID-19 has affected the world of IoT technologies. It looks at the contributions that IoT and associated sensor technologies have made towards virus tracing, tracking and spread mitigation. The associated challenges of deployment of sensor hardware in the face of a rapidly spreading pandemic have been looked into as part of this review article. The effects of a global pandemic on the evolution of IoT architectures and management have also been addressed, leading to the likely outcomes on future IoT implementations. In general, this article provides an insight into the advancement of sensor-based E-health towards the management of global pandemics. It also answers the question of how a global virus pandemic has shaped the future of IoT networks.

## I. Introduction

The Internet of Things (IoT) as a network of sensors collecting data both locally and remotely has proved useful in the field of Electronic-Health (E-Health) management [1]. A combination of Body Area Networks (BANs) and field monitoring devices have allowed for the collection of patient vitals and the provision of track and trace services critical for pandemic management [2]. Locally based E-Health mechanisms can collect health information such as blood pressure, temperature, heart rate, etc. This information can be stored locally and accessed by a health care professional. Local systems can also be used to alert the patient when they need to consult medical personnel and when they need to take medication. Remote based E-Health is essential for health care providers in enabling remote access of patients and patient data. Patient vitals and location can be transmitted at regular intervals to nearby or distant medical facilities for monitoring purposes [3], [4].

In times of a global pandemic such as the 2019 coronavirus (COVID-19), it is critical that social distance guidelines are adhered to and patients are effectively tracked and traced [5]. These two aspects help significantly in controlling the spread of the virus worldwide. The ability of IoT services in providing remote data collection and monitoring of patients in quarantine has made it a critical aspect in fighting the spread of virus pandemics [6], [7].

Health workers and authorities need data to manage a rapidly spreading respiratory pandemic. For COVID-19, data can be used to start the diagnosis of infection and also trace the direction of spread in the community. Primary essential data required includes body temperature, location and travel history [8]. These parameters can alarm officials on whether there is a need for further investigation and testing or not [9]. Initially, health workers resorted to a manual method of measuring temperatures using infra-red thermometers and verbal questioning of people on their history and locations. This posed a risk to health workers due to the increased contact with potentially infected subjects. It had also become an increasingly difficult approach as infection rates reached millions.

Researchers have proposed the use of an already existing network of things to effectively monitor and control the rising COVID-19 infections [10]. A network of heterogeneous sensors existing in form of wearable devices, mobile phones, cameras and drones has fast been integrated into communities. Long-range low-power communication protocols (LPWANs) have enabled coverage of data collection and monitoring over very large areas. As the virus spreads over these areas and across borders, such a feature becomes a valuable monitoring mechanism. Management of thousands of these heterogeneous devices collecting location and temperature data for authorities has further been improved by software-defined wireless sensor networking (SDWSN) [11].

SDWSN [12] in combination with LPWAN technologies such as LoRaWAN [13] has the potential to enable health workers to collect data and monitor the coronavirus spread over multiple locations and large communities. While these IoT technologies are being implemented to combat COVID-19, the inherent IoT platforms and communication protocols are expected to evolve. This is due to the constant adaptations to meet the pattern of virus spread and policy requirements for monitoring and control. Adaptation leading to the evolution of IoT may include modification to the way IoT devices such as mobile phones and drones collect data, changes to IoT management platforms to meet policy requirements and innovations by researchers to tailor IoT technology to effectively manage a virus pandemic.

### A. Motivation of Paper

As the world battles the COVID-19 pandemic that has infected over 11 million people and caused over half a million deaths worldwide [14], there are increased efforts by researchers to find quick solutions towards the effective management of the virus spread. Health-based IoT is increasingly becoming an implementation strategy of choice following WHO guidelines on social distancing and track/trace procedures for infections. Therefore, advancements, adaptations, and ultimately evolution of IoT infrastructure/frameworks for E-Health are expected. We, therefore, see a need to put together an article reviewing the contributions that IoT is making towards the fight against the virus pandemic including the associated challenges. There is also a need to draw a general foresight on the eventual evolution of IoT technologies due to the technical challenges a global pandemic fight provides.

Beginning of 2020 there has been booming research related to IoT-based implementations towards the fight of the COVID-19 and this study aims to integrate these emerging IoT technologies and provide insight on the expected outcomes in the field of IoT deployment. As of date and to the best of our knowledge there has not been a comprehensive survey specifically addressing how the fight against a global pandemic such as COVID-19 is affecting the evolution of healthcare IoT (H-IoT) technologies.

### B. Research Problem and Contribution

There has been rapid contribution in the area of digital technology towards the fight against COVID-19, with comprehensive surveys being provided on the topic in general. However, there is a need for research into the contributions that IoT specifically has made in the fight, the challenges faced and the resultant evolution.

The policies surrounding the need for social distancing on IoT data collection have affected the various IoT implementation strategies. The infectious nature of COVID-19 has led IoT managers to seek various effective and ethical means of sensor deployment. Secure IoT device communication protocols to protect user privacy and maintain system integrity are also expected. Therefore, there is a need to analyze these various contributions and identify potential research gaps.

Additionally, such a research problem when resolved, gives answers to questions like how has IoT been used to combat COVID-19?, how has IoT evolved from before COVID-19 to during the pandemic period? and what are the prospects for IoT in fighting global virus pandemics such as COVID-19? The IoT research community tasked to combat global virus pandemics can benefit effectively from components discussed in such research.

Therefore, this paper provides an in-depth survey of specific IoT-based implementation strategies towards combating COVID-19 and also makes inferences to global pandemic fights in the past. It delves into the various contributions that IoT researchers are making towards tracking and tracing infections and also minimizing the spread of the virus. This together with the associated challenges and resulting IoT evolution. The following is a summary of our contributions.

1) We provide an overview of the H-IoT implementation structure specifically targeted for mitigating the effects of a global viral pandemic. The structure defines the various areas of pandemic management that require H-IoT solutions in particular.
2) We outline several H-IoT implementation strategies towards fighting a global pandemic of which we subdivide based on function and form. We then define an ecosystem for IoT-based COVID-19.
3) We present a review of challenges of pandemic focused H-IoT by detailing a comparison of implementation strategies before and during a global health pandemic. In line with this comparison, we conclude on the resultant evolution of healthcare IoT.
4) Finally, we identify based on the evolution, the future trends of H-IoT in combating and managing a global health pandemic such as COVID-19.

### C. Outline of Paper

This survey is outlined as follows. Section II presents related works from peer-reviewed journals and provides a contribution comparison to our work in tabular form. Section III presents the various IoT components required to effectively combat a rapidly spreading virus such as COVID-19. The section further defines and illustrates how these components come together and form an ecosystem for pandemic management. Section IV begins the discussion on the various contributions IoT is making towards the pandemic fight by first detailing how the technology is being used to manage the critical aspect of social distancing. The discussion on IoT contributions is further continued in Section V where the role of IoT in pandemic data collection and monitoring is presented and emphasized. The concept of the evolution of healthcare IoT in an era of a global pandemic is then analyzed and presented in Section VI. It is in this section that we look at the state of H-IOT before the advent of COVID-19 and also during the pandemic. Section VII delves into the challenges associated with deploying IoT technologies for the benefit of effective pandemic management while Section VIII addresses the expectant trends and available research gaps in implementing H-IoT for combating rapidly spreading viruses in the future. We then set our conclusions in Section IX.

## II. Related Work

The COVID-19 pandemic has become one of the most researched topics since its outbreak in 2019. However, only a few research contributions have discussed the impacts, evolution, and challenges of IoT in the fight against the COVID-19 pandemic. Besides, most available COVID-19 IoT related literature are not published in high-quality and peer-reviewed journals. Here, we have summarized the relevant few related works available.

Siriwardhana *et al.* [15] discussed how 5G and IoT related technologies are developed and deployed to efficiently fight the COVID-19 pandemic. Additionally, some uses cases, as well as challenges on how 5G and IoT can be a candidate for innovative solutions in different areas of the fight against the pandemic, are presented. Similarly, a comprehensive review of the roles major emerging technologies such as 5G, AI, UAVs, IoT and blockchain are playing in mitigating COVID-19 has been presented [10].

Hussain *et al.* [16] highlighted the need to deploy AI in the fight against the COVID-19 pandemic. The study presented an overview of different intelligence techniques that can be deployed for various categories of medical information-based pandemics. The existing AI techniques in clinical data analysis were classified by the authors into neural systems, classical support vector machines and edge significant learning. Finally, a detailed discussion of the advantages of AI in combating similar viruses was presented.

Elavarasan and Pugazhendhi [17] investigated the hidden roles that technologies play in mitigating the COVID-19 pandemic. The study found out that containment strategies implemented alongside innovative technologies had better results in confining society during the pandemic and in controlling the spread of pandemic infections. Additionally, Nižetić *et al.* [18] presented various aspects of IoT with a focus on progress made by IoT technologies in sustainable energy for the environment, IoT smart cities, IoT E-Health-Ambient assisted living systems, as well as in IoT transportation and low carbon products.

Singh *et al.* [7] mentioned the benefits of implementing IoT in fighting the COVID-19 pandemic to include reduced healthcare cost and improved treatment outcome of the infected patient. The study highlighted the application of the IoT philosophy roadmap in tackling the COVID-19 pandemic by identifying and discussing twelve (12) significant applications of IoT. Furthermore, Mbunge [19] analysed the potential opportunities and challenges of incorporating emerging technologies for COVID-19 contact tracing. In a related study, Javaid *et al.* [20] presented a detailed review of the application of industry 4.0 technologies in the fight against COVID-19.

The application of drone-based technology in the fight against COVID-19 was presented by Kumar *et al.* [21]. Additionally, an architecture for tackling pandemic related emergencies in several scenarios using real-time and simulation-based scenarios was also proposed. Vaishya *et al.* [22] identified seven (7) significant applications of AI for detecting cluster cases of COVID-19 infections as well as for predicting future incidences of infections by collecting and analyzing previous data. To summarize the related works, in Table 1 we present the main differences of the cited related works in comparison to our survey. The major contributions of our survey addresses the identified research gaps while complimenting the contributions of existing related works.

**TABLE 1:** A Comparison of Related Works on Aspects of IoT Based COVID-19 Management

Reference	IoT based COVID-19 aspects				
	IoT as objects/infrastructure in smart cities for fighting COVID-19	Discussion on data collection/sharing (models and challenges for COVID-19)	IoT solutions for social distancing	COVID-19 H-IoT evolution	IoT challenges for COVID-19
Siriwardhana et al. [15]	✘	✔	✔	✘	✔
Chamola et al. [10]	✘	✔	✔	✘	✔
Hussain et al. [16]	✘	✔	✘	✘	✘
Elavarasan et al. [17]	✘	✘	✔	✔	✔
Nižetić et al. [18]	✔	✘	✘	✘	✔
Singh et al. [7]	✘	✘	✘	✔	✔
Mbunge [19]	✘	✘	✔	✘	✔
Javaid et al. [20]	✘	✘	✔	✘	✘
Kumar et al. [21]	✘	✔	✔	✘	✘
Vaishya et al. [22]	✘	✘	✘	✘	✘
This survey	✔	✔	✔	✔	✔

## III. Healthcare IoT for Virus Pandemic Management

Deploying IoT technology in fighting a global virus pandemic results in a well-defined ecosystem of hardware, software and associated policies. This section delves into defining the components of this particular healthcare ecosystem based on findings from surveyed research. The ethos of using IoT based technology to manage a pandemic produces a niche set of well-integrated components. These components work together as part of an ecosystem driven to fight and minimize the spread of a virus. Other advantages of using IoT to fight a pandemic such as COVID-19 include higher accuracy for patient management, reduced cost, effective control, effective diagnosis and provision of opportunities for superior treatment [7]. An ecosystem such as this can be divided into the following main components as defined by various research contributions.

### A. Sensor Hardware

Deployed sensor hardware is a critical aspect of H-IoT infrastructure. Such hardware makes for a primary source of data that can be used to drive the purpose and goals of the IoT implementation. In fighting and managing a virus pandemic, sensor hardware can take the form of wearable devices suitable for BANs and or smartphone-based using inbuilt sensors such as geolocation chips, accelerometers, cameras, etc.

Albezuirat *et al*. [23] envisioned the need to use IoT devices to monitor the spread of the pandemic for immediate case reporting. The deployed smart devices could be used in airplanes, airports and also be made available to individuals by implementing ease of use in the hardware design. To facilitate pandemic management while observing social distancing, a network of computers and smartphones is essential in providing virtual communications [7].

Low-cost hardware such as this has been driven by the need for rapid screening while maintaining a safe distance [24]. A popular option for low-cost sensor hardware in fighting a pandemic is the use of already existing smartphone hardware. The smartphone accelerometer, microphone, camera and temperature sensors are used in combination with machine learning (ML) algorithms to detect early COVID-19 symptoms. Geolocation sensors and drone technology are also being used in the detection and diagnosis of COVID-19. Mohammed *et al*. [25] proposed a thermal imaging IoT drone mechanism to detect suspected high body temperatures due to COVID-19. Recently, a similar detection algorithm using a smart helmet as the primary hardware has also been proposed [26]. The smart helmet uses thermal imaging cameras and geolocation tags to detect and report suspected COVID-19 fevers.

### B. H-IoT Based Software

Various software and management tools are being developed to better manage sensor data collection and filtration. Major focus areas include energy efficiency, lightweight mechanisms, efficient and less intrusive data collection techniques among others. Recent software implementations tend to focus on making the most use of already existing hardware such as smartphones to form part of the H-IoT ecosystem.

The first line of software solutions in tracking and tracing infections has been demonstrated by android and ios mobile phone operating systems. Both systems have integrated APIs to alert the user on a potential COVID-19 exposure risk [27]. Using embedded smartphone proximity sensors the API provides an opportunity to alert a user if someone near them recently tested positive. Governments in India and Singapore have promoted the development of smartphone applications to ensure effective tracking and tracing of people that have tested positive [28], [29]. Researchers however are still debating on the efficacy of using smartphone applications for contact tracing against the privacy of patient data [30], [31]. As part of a campaign for global monitoring of the virus spread, the required increased computing power by IoT devices is being provided by SDN and cloud services [4], [24]. Furthermore, to limit interaction between patients, healthcare givers and IoT managers; virtual communication tools are being developed leading to the implementation of virtual clinics [32].

### C. Data Extraction and Analysis

Sensor infrastructure in the IoT field produces essential data that meets the goals of an IoT implementation towards managing a problem. To better manage a pandemic researchers need information on infection and patient status, and location data for track and trace purposes. However, with increased sensor deployment we begin to see data accumulation leading to the need for useful data filtration and big data management mechanisms. The use of machine learning techniques and artificial intelligence in general is becoming a popular route in the analysis and decision-making process of sensed data.

Machine learning algorithms have been used for training thermal images depicting both negative and positive COVID-19 tests [26]. Elavarasan and Pugazhendhi [17] identity artificial intelligence and machine learning in combination with IoT as a viable combination of technologies in fighting a virus pandemic. Deep learning a component of artificial intelligence can effectively be used to diagnose the COVID-19 infection based on CT scans and x-ray images with minimal error [33].

Another important data analysis feature being used in tackling the spread of the corona-virus is trend analysis in combination with machine learning. COVIDSens [34] uses social media feeds and machine learning to track and trace virus propagation. Based on user experiences posted on social media, the application can filter useful new information for the benefit of the government and the general public. We also envision opportunities for using IoT virtual sensor nodes and machine learning to determine missing data and predict future infection rates.

### D. Regulatory Framework

Sensor deployment in the field and on the human body needs to adhere to ethics and regulations. The design communication radio frequencies and channels should be safe for device placement close to the body. Considering the risk of virus infection there is also a need for regulations on the interaction between patients and H-IoT system managers. Another hot issue is related to the need for standardization of protocols for increased data sharing among communities. There is a growing demand for people to provide location and travel history in exchange for virus spread control and management. How do IoT managers ensure patient data privacy and security? These are critical ethical issues that need to be addressed.

Allam and Jones [5] present an elaborate perspective on the standardization of data sharing in a smart city network as the world battles COVID-19. There is an urgent need for standardization of communication protocols in smart cities. This is to encourage fair usage and transparency of user data in times of a global pandemic. There remains numerous ethical questions regarding data collection by IoT devices for pandemic management. Questions on how secure, ethical and effective these user data collection methods are? arise. As part of the regulatory framework, researchers are calling for government entities to assure the public on the safety of their data [30], [31], [35].

Gasser *et al*. [36] discuss digital tools being used in fighting COVID-19 in relation to features and ethical concerns. They also offer a navigation aid towards a satisfactory regulatory framework for policymakers. The proposed aid ensures satisfactory approval and governance of IoT digital tools developed towards fighting a rapidly spreading virus while allowing for risk-benefit optimization [36]. Guidelines such as those provided by GSMA [37] are necessary for stakeholders to ensure trust and security of user data. All this while providing the much-needed help towards the fight against a pandemic.

**FIGURE 1.**
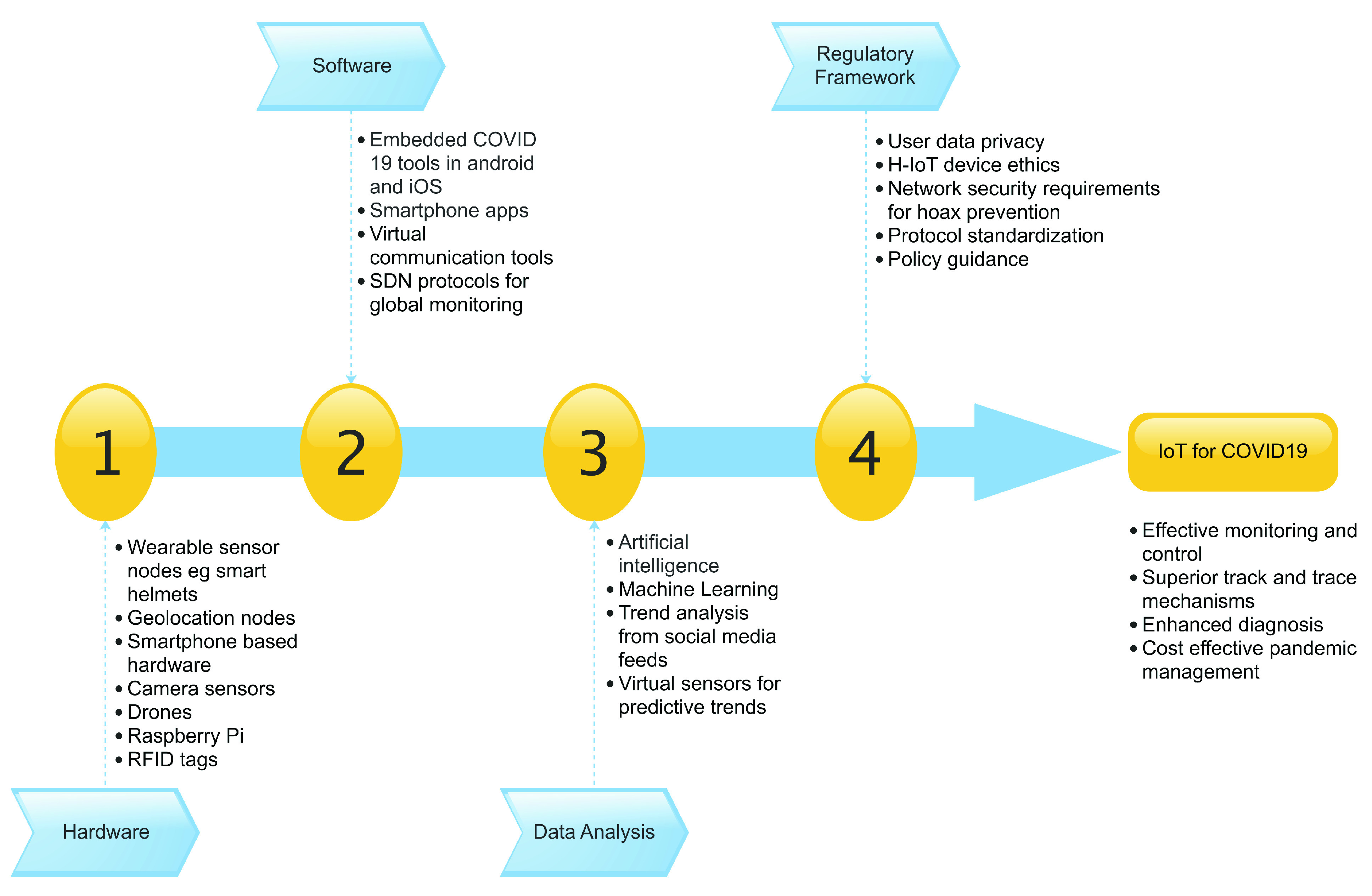
IoT for COVID-19 ecosystem.

The IoT components discussed thus far are illustrated in Fig. 1.

## IV. IoT Solutions for Social Distancing

Social distancing plays an important role in limiting the spread of the virus together with countrywide lock-down and reduction of the workforce to essential workers only. New IoT technology to integrate social distancing in managing a global pandemic has been a major contributing factor to the evolution of IoT. We are now seeing state-of-the-art IoT systems using AI in combination with social media posts to warn communities to avoid high-risk areas [34]. Often when there is a breakout in a particular area, people tend to share reports and opinions on social media, mentioning locations and statistics in question. This information in combination with a suitable AI algorithm can produce vital social distancing data for affected communities. We envision this warning data to be integrated with embedded geolocation sensors in user smartphones as a provision for notification whenever one is near a reported high-risk area.

Drones are being used in Spain and other countries with loudspeakers reminding the population to stay at home [38] and also for thermal imaging purposes. Drones can collect data on crowd gatherings for authorities to monitor and enforce social distancing guidelines. Data collected here can also be fed into smart city and Intelligent Transport Systems (ITS) infrastructure. This aspect can be accelerated by the implementation of IoT as objects and infrastructure.

Other IoT applications focus on implementing virtual distant meetings on smart devices. The culture of work from home is strongly being enforced using a network of smart devices with installed applications such as Zoom cloud meetings, Google meet, Microsoft teams, etc. [39].

Economical wearable smart devices are also being proposed that allow the use of sensors outside the smartphone to alert users on social distancing [40]. Extra features such as thermal screening of people within close range of the user have also been added as part of the smart device. Generally, the proposed device creates social distancing awareness for the device wearer by providing notifications whenever there are people nearer than the stipulated social distance guidelines.

While the above applications are proving useful, they do not come without setbacks and limitations. Privacy concerns and the reluctance of the public to share data remain a huge challenge. The use of smartphone applications, drones, and ITS infrastructure has been faced with drawbacks concerning privacy. Security and anonymity of data collected are raised by stakeholders whenever such IoT deployments are made. Data accuracy and reliability is another challenge that has to be overcome by IoT related technologies such as AI and big data. This, because of the heterogeneous data sources involved in managing a global pandemic. The use of social media data approaches is no exception to this problem. Finally, user safety concerns remain an issue with wearable e-health devices.

A summary of the social distancing mechanisms detailed in this section is shown in Table 2.

**TABLE 2:** IoT Technologies Implemented Towards Enforcing Social Distancing

S. no	Social distancing measure	Description	Application use-cases	Challenges
1	Social media approaches	Based on using data from social media posts in combination with AI.	COVIDSens [34]	High chance of false positives due to hoax social media posts.
2	Smartphone applications	Usually involves the installation of a tracker application on smartphone. Users can be notified if they are near a potential risk patient.	TraceTogether [41], Corona-warn-app [42], Aarogya-Setu [43].	Privacy concers are still a huge challenge.
3	Drones	During pandemics drones can be used to deliver goods and services. Drones can also be used in combination with a loudspeaker to propagate social distancing guidelines to communities.	India [44], Spain [38]	Device travel range and battery life still remain critical. Privacy concerns regarding being monitored by drones.
4	Smart city and ITS	Data-driven approach using AI and data provided by smart city and ITS infrastructure to guide traffic and minimize large crowds through smart scheduling.	Smart city drone monitoring [45], smart packing and traffic re-routing.	Security, privacy and policies regarding the collection of data from public infrastructure.
5	AI and Big data	IoT hardware deployed in communities and also embedded in smartphones can provide essential big data necessary for developing social distancing AI systems.	Crowd hot spot identification in a city. Tracing and limiting movement of infected individuals with the general population [46]	May require large amounts of classified and labeled data to be effective.
6	Remote meetings	Providing an alternative to meeting in person against social distancing guidelines. This approach makes use of a network of smart IoT devices and application packages to enable virtual meetings from multiple locations.	Zoom, Microsoft Teams, Google Meet, Cisco Webex [39]	Requires a solid internet connection and users need to familiarize themselves with the application packages.
7	Wearable smart devices	This involves wearing a smart device to provide localization and tracking of people around the user. Thermal sensing technology can also be incorporated to detect potential COVID-19 symptoms in a crowd.	Crowd and risk warning systems [40]	Ethical and safety requirements of wearable health devices.

## V. Data Collection and Monitoring

The foundation of most IoT deployments including one intended to combat a virus pandemic is the aspect of monitoring and data collection. Sensors placed in airports, other ports of entry, worn on the body, embedded in devices play the important role of collecting data necessary for input in a pandemic management system. The data collected is a key aspect for enabling authorities to effectively monitor and control the spread of a virus. The various IoT mechanisms being used in data collection and monitoring of the COVID-19 virus will be addressed in this section.

### A. Data Collection

To discuss the data collection aspect with enough clarity we need to identify the information required in managing the spread of a virus pandemic such as COVID-19. The following parameters have been identified as essential for first-line detection and tracking. These data parameters are collected by IoT devices in the field and fed into IoT monitoring platforms.

1) Temperature: High fever is a major early symptom of COVID-19. Therefore, temperature checks are critical in detecting a potential infection. Several mechanisms for temperature collection are being promoted for being non-touch and within acceptable social distancing measures. Examples include non-contact infrared thermometers [47], thermal imaging cameras, smartphone cameras, robot mounted temperature scanners [48] and wearable thermal sensors [26]. These temperature devices can be deployed as part of an IoT framework sending readings to an applicable monitoring system. A higher than normal temperature reading should trigger an alert for further investigation throughout the health management system.
2) Location: Where have you been? Where are you currently? These are two crucial location queries for effective tracking and tracing of potential and infected COVID-19 patients. Geo-location sensors are being used to track the location of individuals to limit the risk and spread of the virus. These location devices can take the form of smartphone GPS [49], wearable location trackers for positive individuals [2], ITS infrastructure cameras spotting your location, etc. Mobile phone companies are also being used to provide anonymous location data as part of the fight against the virus spread [50].
3) Imaging: Computer vision is not only being used for thermal imaging in the fight against COVID-19 but it also has the potential of identifying trigger images. These trigger images may include crowds breaching social distancing guidelines and the presence of a quarantine subject in a public area [51]. CCTV and drone footage can provide a source of video surveillance data necessary to limit virus spread by human presence in certain areas [20], [36].
4) Pay-point data: To further enhance the efficiency of track and trace mechanisms, credit card transactions have been proposed to be tracked. Data from pay-points can give information on time and location a transaction was performed by a quarantine subject. This novel mechanism however is greatly hindered by privacy concerns [52]. In South Korea, the success of this mechanism is said to enable tracking of patient location within 10 minutes [53].
5) Social media feeds: AI techniques such as COVIDSens [34] can extract posts and sentiments from social media as input data and provide a simulation of the COVID-19 virus spread. Using feeds from platforms like Twitter and Facebook, information on social distancing, virus hotspots and individual tracking can be extracted. The nature of this data is such that it is possible for fake news to be posted and therefore extensive trend analysis and big data analytics need to be implemented. AI can improve the accuracy of the data being provided by social media feeds.

### B. Monitoring Systems

The data collected by IoT sensory systems can get very large and therefore a suitable framework is required to produce the expected outcomes of the technology. There is a need for intelligent systems to analyze and monitor the spread of a virus pandemic based on the provided data. Consequently, this brings in the aspect of big data analytics, AI and visual mapping for IoT infrastructure frameworks.

1) IoT big data and AI: Big data analytics and modeling can enable real-world simulation of the coronavirus spread. Several researchers are using artificial intelligence to better analyze COVID-19 big data and enable effective track and trace mechanisms [34], [46], [54]. Naude [55] mentions in an early review that AI is a powerful tool in the fight against COVID-19 and discussed several use-case scenarios for pandemic detection, tracking, tracing and spread prediction. On the aspect of big data analytics, most of the value in terms of pandemic combat remains as a future prospect. The major reason lies in community acceptance and willingness to share data. The explosion of thermal imaging data, location data, social feeds, etc. creates a good opportunity for data scientists to monitor the virus spread and provide solutions for health management.
2) Visual mapping: Development of real-time data dashboards simulating the spread of the virus worldwide. Dong *et al*. [56] develop one such dashboard with an interactive web-based interface. Dashboards rely on artificial intelligence and multiple data sources including location data, social media feeds and government information. A visual representation of the state of the virus with a graphical representation of actual and predicted numbers are provided within such AI-based dashboards. Visual mapping can rely on the IoT framework while existing in the application plane for SDN-based deployments.

As standalone mechanisms, the data collection methods discussed in this section are not sufficient to effectively mitigate and manage the spread of COVID-19. Deployment of each method comes with a challenge that needs to be augmented with another method or technology. Screening of COVID-19 based on temperature, for example, does not confirm that one has been exposed but provides a means for further investigation. Location and imaging can help determine whether one has been in a high-risk area however, a hindrance exists regarding data security and privacy. People are not fully ready to share their location and images for the benefit of pandemic management. Another security-related concern is the aspect of using pay-point data to determine location. What if authorities are using the data for more than just location monitoring? Who else is snooping on this data? These are some of the privacy questions that arise with such mechanisms of data collection. Social media is proving to be a source of abundant and quick data but it is prone to people posting fake news and hoaxes.

A summary of the above data collection and monitoring mechanisms is presented in Table 3 with associated challenges.

**TABLE 3:** Summary of Data Collection and Monitoring Systems for IoT Based COVID-19 Management

Data parameter	Extraction technology	Monitoring system	Challenges
Temperature	Non-Contact thermometers, thermal imaging, smartphone based thermal imaging, robot and drone mounted, smart helmets	AI based image processing, Big data, visual dash boards.	Temperature alone is not sufficient basis for COVID-19 diagnosis.
Location	smartphone location tracking, wearable trackers, smart city and ITS.	Mobile phone company AI algorithms, machine learning algorithms embedded in smartphone apps, ITS big data, visual dash boards.	Major privacy concerns
Imaging	Public CCTV and drone video surveillance, ITS based cameras	AI based image processing and identification	Mistaken identity, privacy concerns.
Pay-point data	Location data loggers, camera footage at pay-points	Alert systems flagging user location, visual dash boards, location mapping.	Transaction data security, privacy concerns.
Social media	Twitter, Facebook	AI character recognition flagging keywords in social posts, Machine based data filtering applications	Unreliable information and fake news.

## VI. Evolution of Healthcare (Pandemic) Based IoT

In the aftermath of World War II, several research efforts were invested into ‘urban intelligence’ to bring scientific principles into urban activities [57]. Consequently, several of those efforts led to what is today known as the concept of ‘smart city’ [57], [58]. Indeed, the concept of urban intelligence outlines three essential know-hows comprising city information resources, scientific skills in data handling and executive power for readiness and response in the event of a pandemic [57]. For instance, skills such as data sensing and mining as well as integration, modeling, analysis, and visualization of information have been used in responding to a pandemic. Basically, urban intelligence describes the application of data science frameworks and computational techniques in solving domain-specific urban challenges.

In recent times, research activities in the field of the Internet of Medical Things (IoMT) have proposed a new paradigm in the healthcare system known as smart healthcare or intelligent healthcare (healthcare IoT). Research endeavors into healthcare IoT were driven by the earlier research and development efforts in wireless sensor networks (WSN). Primarily, IoMT deploys ubiquitous internet-enabled digitally connected devices with embedded identification, sensing capabilities and data exchanging features. This is to bridge the gap between a patient and the healthcare givers. Certainly, the objective of intelligent healthcare is to utilize innovations in the management of the healthcare system. For example, intelligent healthcare deploys wearable gadgets, versatile web and IoT to gather data of individuals, equipment and agencies associated with healthcare services and then it uses this information to superintend and respond to healthcare needs in an insightful way [59].

Take COVID-19 for instance, intelligent healthcare can contain the transmission and spread of the virus by collecting, integrating and analyzing precise, relevant and high-quality data in real-time. Similarly, intelligent healthcare can collect data via patient-centered health-based apps to track new COVID-19 cases. Also, wearable tech (body-worn-sensors) can be deployed to administer healthcare to COVID-19 patients through continuous connected care instead of encounter-based care. Additionally, potential COVID-19 hotspots can be proactively identified and monitored through continuous data stream and development. This facilitates the prevention and spread of the virus. Also, intelligent healthcare can improve community safety through the integration of different data sources [60]. For a better understanding of health-based IoT, we review the current trends in the industry and how it is applied to healthcare. Based on our findings, we have separated the IoT-based healthcare evolution into two categories. Firstly, the application of H-IoT in the pre-COVID-19 era. Secondly, how healthcare IoT has responded to the outbreak of the COVID-19 pandemic.

### A. Research and Development Efforts in Healthcare IoT Pre-COVID-19

Healthcare IoT is capable of connecting and integrating patients, medical personnel, body-worn sensors, medical things and information technology systems through on-demand internet connectivity [60]. The deployment of H-IoT has potential benefits for increased workforce productivity, operational efficiency as well as improved patients’ experience. These benefits eventually led to cost savings and reduction in human error. In terms of design and implementation, H-IoT devices have physical properties that largely define their implementation characteristics. These properties include the following.

• Tracking
• Identification and authentication
• Data collection and monitoring
• Sensing, control, automation and optimization

Given this, in the pre-COVID-19 era, the application of healthcare IoT has largely been patient-centered characterized by the following factors.

1) Safety: Perhaps the most auspicious benefits of the deployment of the H-IoT pre-COVID-19 era is patients’ safety. Healthcare IoT ensures patients’ safety by remotely connecting healthcare services with patients who need such care through real-time healthcare data management [57]. Real-time health data management guarantees the patient’s safety by remotely monitoring and predicting the patient’s vital signs and real-time health status. Similarly, H-IoT provides pharma-epidemiology and extensive population health management services for patient’s safety. Also, in a pandemic scenario, healthcare IoT enables public health policy and regulation decisions to guarantee public safety [57].
2) Satisfaction: Healthcare IoT takes primary or secondary healthcare interaction away from the traditional encounter-based care to interactive virtual healthcare through connected continuous care. The convenience of accessing telemedicine and responsive feedback between patients and healthcare givers influences patients’ satisfaction positively [57].
3) Engagement: H-IoT coordinates healthcare decisions (care coordination), manages prescription (medicine adherence), and create patient-specific education (personalized healthcare and well-being planning). This has been possible through health data management by interactive virtual reality [20].

### B. Research and Development Efforts in Healthcare IoT During a Pandemic: A Case of COVID-19

Besides the recent novel coronavirus which started at the end of 2019 in Wuhan, there have been three deadly pandemics over the past century in 1918, 1957, and 1968 [58]. Generally, a pandemic is described as a disease that spreads fast in several countries and continents resulting in an unprecedented social and economic impact on society. Moreover, current globalization caused by rapid urbanization, population growth and increased global travel has made many cities around the world a hub for rapid transmission of the pandemic. However, the adoption of smart technologies e.g. IoT-based healthcare, big data, and AI has made cities more resilient in curtailing the spread of a pandemic [58].

In the wake of the COVID-19 pandemic, for instance, most cities around the world have adopted either a human-driven or techno-driven approach in the fight against the pandemic. The techno-driven approach mandates cities and their citizens to adopt smart technology by imposing technologies on them using an enhanced top-down method. However, the human-driven approach encourages cities to adopt smart technologies required in the cities by educating its citizens and enhancing their potentials.

China’s techno-driven approach which has been lauded by the WHO-China Joint Mission report as “the most ambitious, agile and aggressive disease containment effort in history” has largely been effective in fighting the pandemic [58]. However, the human-driven approach adopted by countries in Europe and the USA has had a lower impact on the spread of the pandemic. Besides, the human-driven approach adopts a soft technological determinism stance which gives citizens the freedom to choose the technology they want to use. On the other hand, the techno-driven approach employs a hard technological determinism stance where technology is considered as a tool in solving problems encountered by governments.

**FIGURE 2.**
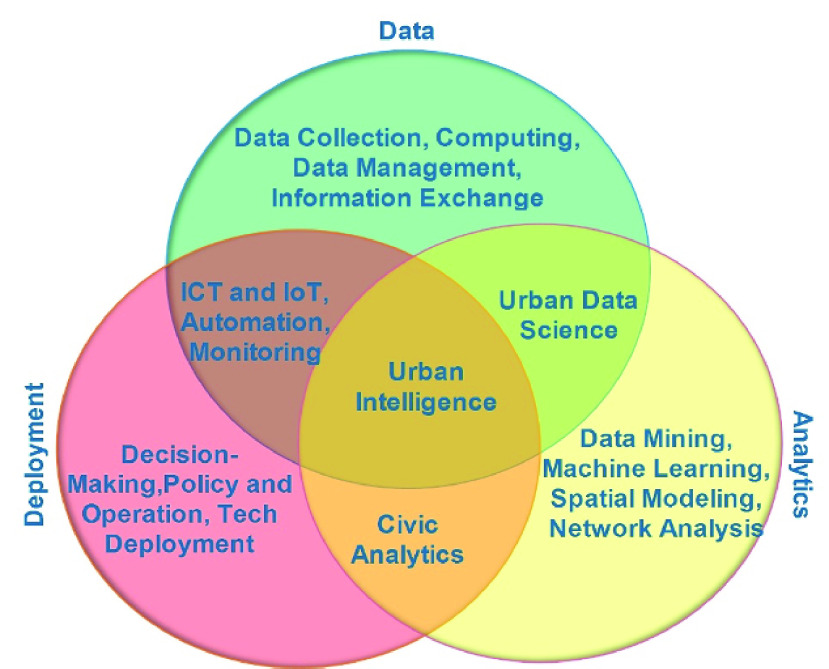
Core components of urban intelligence. Adapted from [57].

The help of IoT, big data and AI, as well as the innovative application of healthcare IoT in smart cities across China, Europe and the USA has aided significantly in the fight against COVID-19. The technology has enabled continuous monitoring and prompt decision making. Fig. 2 shows the core components of urban intelligence necessary in response to a pandemic [57]. Similarly, there is a commitment now with a greater reason for the advancement of different cutting-edge technologies to tackle various challenges related to this virus pandemic. We present how different technologies in healthcare IoT have evolved towards the fight and management of the COVID-19 pandemic.

#### 1) IoT Platforms and Communication Protocols

To ensure the presentation of useful COVID-19 information processed from raw IoT data, researchers are developing the front end of IoT platforms to have dashboards detailing statistical COVID-19 related information. The potential impact of IoT platform dashboards in the fight against COVID-19 has been emphasized in a commentary by James *et al.* [61]. Smart city IoT data and analytics infrastructure have been cited to provide real-time insights on COVID-19 and the impact it has on urban governance. IoT dashboards are rapidly being implemented as part of an urban observatory to capture and display results of city interventions to combat COVID-19. Dashboards have also been reported to show insights on the environmental impact of planned social distancing measures by authorities with improvements in air quality being observed [61].

Hossam *et al.* [62] propose an integrated IoT platform that takes big data from multiple IoT sensors as input and produces information on an interactive dashboard. The IoT platform proposed by the authors consists of three subsystems namely, an embedded microcontroller, an IoT network and an AI system. The microcontroller is used in tandem with the IoT subsystem to collect user data for the AI subsystem to analyze and forecast the virus spread rate. The AI system also updates front end users on the status of the virus in a particular region through a dashboard map.

Otoom *et al.* [63] have recently proposed another IoT framework for early detection and monitoring of COVID-19 cases. The proposed framework consists of five main components namely the sensor hardware (wearable devices), the quarantine center, the data analysis component utilizing machine learning, health workers and the cloud platform. The platform uses eight (8) ML algorithms to effectively and quickly identify potential COVID-19 cases. Five of these algorithms were found to have an accuracy of more than 90%.

As regards IoT communication protocols, we found from the literature covered that researchers are relying on the already existing IoT communication technologies such as 6LoWPAN, LPWANs and Bluetooth to relay sensor data to IoT management layers or subsystems [62], [63]. In terms of network lifetime and widespread deployment of sensors to collect COVID-19 data, LPWANs provide the necessary long-range low-power communication protocols to support the IoT framework. LoRAWAN can be used to periodically send patient data over large distances while maintaining device battery longevity.

A multi-agent approach to combating COVID-19 using multiple LoRaWAN devices has been proposed to monitor sanitation levels and other COVID-19 related data at an airport facility [64]. The focus of the project was ensuring high sanitation levels in airport toilets during this COVID-19 era. Away from combating COVID-19 infections, other researchers are interested in the resultant environmental impact of COVID-19. Howerton and Schenck [65] propose and implement a LoRAWAN based IoT framework to monitor air quality before and during the COVID-19 outbreak with results showing a general decline in carbon dioxide levels.

However, as virus cases and IoT for COVID-19 deployments rapidly rise, there is an expected influx of data being sensed. This would require high-speed transmission of data to the subsystems tasked to analyze and manage COVID-19 data. This leads to improved accuracy and efficiency of performance. 5G for IoT has been poised to provide this high-speed low latency communication protocol for IoT based COVID-19 management [10], [66]. This has further been facilitated by the drive to push 5G infrastructure as part of the IoT framework for smart cites [67]. Such infrastructure can enable high-resolution thermal images to traverse through the IoT framework at high speed thereby keeping up with monitoring the rapid virus spread.

#### 2) Drone Technology for IoT

Drones can be deployed in tracing the outbreak of COVID-19 including tracking of persons who came in contact with COVID-19 patients [68]. Similarly, drones are helpful to enforce and track patients who breach quarantine as well as ensure adherence to wearing face masks [58]. For instance, in Hubei including Europe and the USA, drones were used to ensure that lock-down and social distancing rules were strictly adhered to by residents [58].

Similarly, drones equipped with cameras were deployed to issue instructions and warnings to residents not wearing a face mask or breaking emergency protocols [58], [69]. Also, drones can be used in remote monitoring of in-home patients or highly infected areas. For instance, drones have been deployed to deliver life-saving materials to healthcare givers as well as collect and transport samples for testing in nearby facilities.

#### 3) H-IoT Based Artificial Intelligence

AI can be deployed to evaluate the risk of infection as well as to screen residents. Moreover, with AI, systems can be instructed to use models based on big data to recognize, explain and predict a pattern and generate actionable awareness [60]. For instance, AI applications have been used to monitor and report the travel history of residents from highly infected areas to the appropriate authorities [58]. This is particularly helpful in predicting the outbreak of the virus, in addition to minimizing or stalling the spread of the virus. Similarly, with lots of misinformation regarding the virus on social media, AI-based systems can be trained to remove wrong information from social media. Furthermore, optimized clinical trials of drugs and vaccines are possible with the deployment of AI [58]. Also, AI has been used to build robots that are capable of performing online medical examination/AI-assisted diagnoses of residents as well as disinfecting and sanitizing the environments.

In China, for example, AI-assisted CCTV cameras with face recognition capabilities were installed in apartment doors to ensure that residents adhere to quarantine rules and do not leave their homes. Also, decentralized testing was conducted across Chinese cities using AI to identify COVID-19 infected residents. Moreover, Organizations e.g. Megvil technology limited, Baidu, SenseTime have each developed AI-assisted contactless body temperature screening system which can be installed in public places to identify those infected with COVID-19 [58]. Specifically, AI-assisted systems with contactless remote temperature screening can screen about 15 people per second from a distance of 3m. Generally, AI systems have been beneficial in manufacturing equipment required to fight the COVID-19 pandemic. Also, it has provided relief for the overstretched health care systems [20], [58].

#### 4) H-IoT Sourced Big Data

Undoubtedly, a huge amount of data is collected by IoT sensors, from social graphs, smartphones, and public data from the transport, environment, and healthcare sectors [5]. As a result, there is a need to coordinate, manage, analyze and understand all biomedical big data collected. Sources of big data include medical clinics, recovery/isolation centers, ubiquitous wearable devices, networks and homes [17].

**FIGURE 3.**
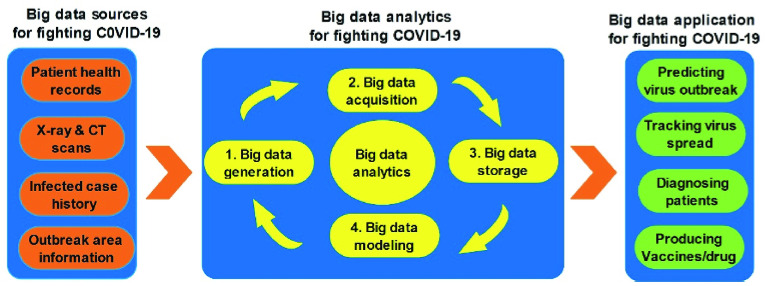
Application of big data in the fight against COVID-19. Adapted from [70].

Potentially, big data can support healthcare IoT by using intelligent analytics to exploit electronic healthcare records in healthcare domains including patients’ health analysis, diagnosis support and drug support [70]. In the fight against the COVID-19 pandemic, big data has been useful in four main fronts as shown in Fig. 3 which include: prediction of a virus outbreak, tracking of virus spread, diagnosis/treatment of patients, and discovery of vaccines/drug.

Typical IoT-sourced big data uses in fighting the COVID-19 pandemic include:

• Outbreak prediction: Big data supported by AI and predictive analytics have played a vital role in predicting the outbreak of the COVID-19 pandemic [71]. For example, AI-based systems such as BlueDot and Metabiota have been used to detect initial signs of COVID-19 [72]. Similarly, the WHO has acknowledged the application of big data and AI in COVID-19 containment measures. Furthermore, WHO admits that without the role of these cutting-edge technologies, the spread of COVID-19 would have been faster and overwhelming.
• COVID-19 tracking: As the COVID-19 pandemic continues to ravage the world, real-time data is constantly being collected from different sources around the world using COVID-19 trackers. As a result, epidemiologists, scientists, doctors and policymakers are equipped with the latest information to make adequate and responsive policies to prevent the spread of the disease [20]. Particularly, areas affected by COVID-19 are displayed on the system and strictly monitored using big data analytics [71]. For instance, human movement patterns from previously infected locations are analyzed to identify, track and predict the outbreak of the disease in probable locations. Take the UK for example, data collected by the National Health Service (NHS) telephone networks were analyzed in partnership with Amazon, Microsoft, and Hancock to move available resources to tackle the pandemic. In China, local governments were permitted by the China Health Commission to use AI-assisted big data to model, estimate and to track the COVID-19 pandemic in real-time [70].
• Patient diagnosis: Biomedical big data collected from several COVID-19 big data sources have been used to establish a cloud-based system for the diagnosis and treatment of COVID-19 patients. For example, several clinical tests and analyses including typical and atypical CT/X-ray imaging manifestation, hematology examination and pathogen detection have been carried out on a large-scale. This with data comprising of 11,500 persons from Zhongnan Hospital of Wuhan University to diagnose and treat COVID-19 [70]. This data is shared and studied by experts to understand the pathogenesis, structure, and therapy of the virus. Similarly, basic clinical physiologic and medical imaging information of each patient are being analyzed for accurate diagnosis and scientific medication [73].
• Drug and vaccine production: Electronic health records (EHRs) of COVID-19 infected patients collected from a variety of real-world sources have facilitated a large-scale investigation. This has been used to develop comprehensive treatment solutions with high reliability using big data analytics. For example, the spike protein and structure of SARS CoV, MERS CoV, SARS-COV-2, and other human coronavirus strains have been investigated to develop suitable COVID-19 vaccines [70]. Similarly, the National Center of Biotechnology is facilitating vaccine production from big data through big data analytics [9]. Furthermore, machine learning assisted big data-aided drug repositioning system which combines knowledge graph and literature to develop the COVID-19 vaccine and manufacture COVID-19 drugs have been developed using big data analytics.

#### 5) Cloud Assisted IoT

With advancements in wireless and digital technologies, computer system resources e.g. database, networking, servers, and intelligence can today be delivered over the internet [20]. Cloud computing provides faster and flexible resources and innovation as well as the cost-effective and efficient running of infrastructure. During the COVID-19 pandemic for instance, while most people were isolated from their normal lives they have been able to continue their digital lives thanks to applications such as Zoom video, Google Meet, Google Cloud, Slack, Amazon Web Wervices, Netflix, and Microsoft Azure. Similarly, healthcare workers have been able to manage a large volume of requests arising from the COVID-19 pandemic with healthcare-specific apps like Salesforce Care solution [20].

H-IoT devices in the field operating with limited resources such as energy and computing power have benefited from cloud services. With concepts like cloud computing, moving energy and other resource heavy tasks has been possible. Ultimately, sensor nodes are mainly left with the task of collecting COVID-19 data and transmitting it to the cloud. Therefore, energy of these devices is mostly consumed during transmit and receive sessions of antennas. This combined with suitable energy saving algorithms can prolong the lifespan of the IoT devices.

**FIGURE 4.**
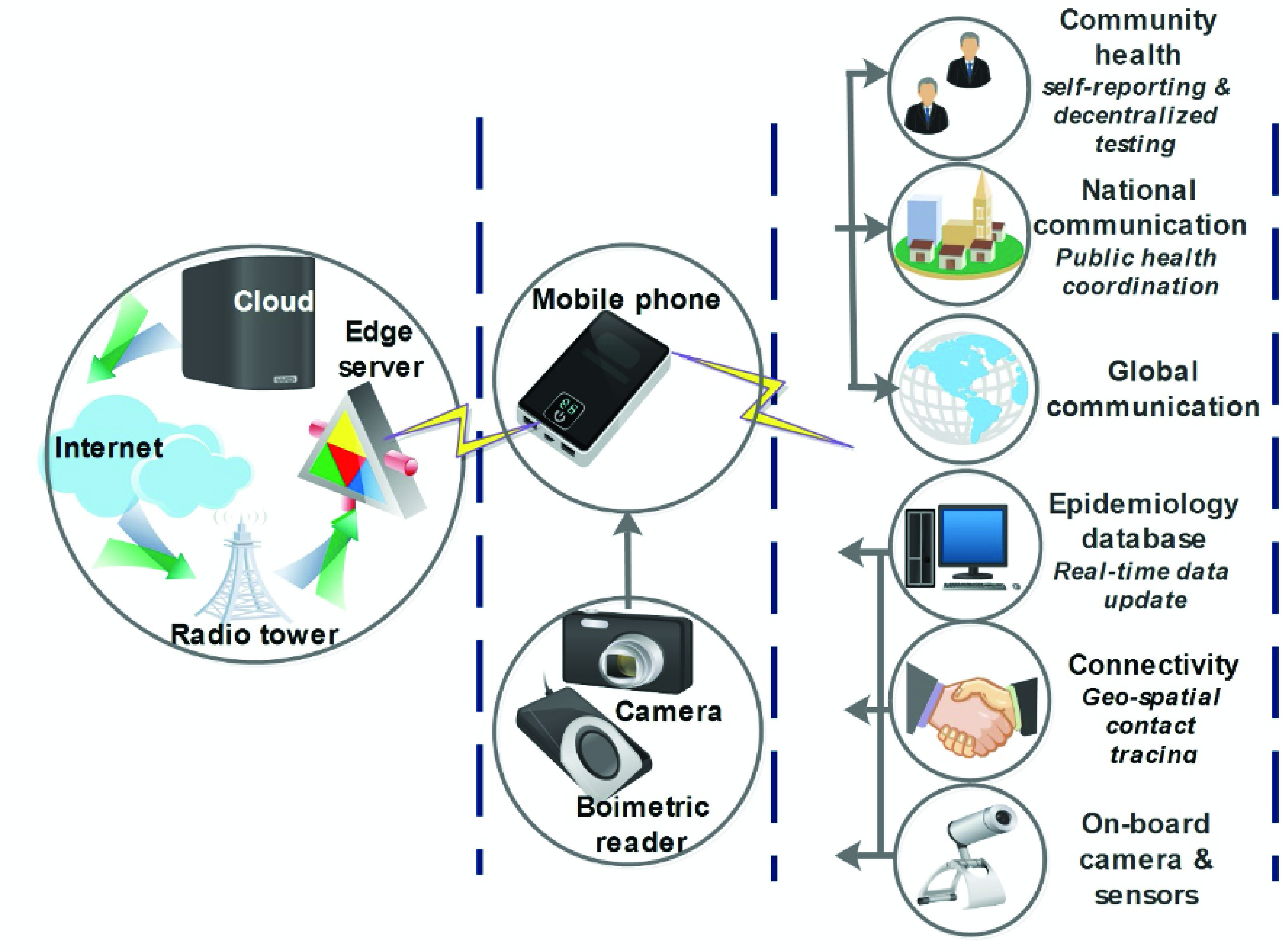
IoT and cloud-based framework for fighting COVID-19. Adapted from [70].

In smart cities across Europe and China, mobile phones with embedded cameras and biosensors have collected personal information e.g. X-ray, CT images, heart rates, and cough sounds which are then encrypted, compressed and sent to the cloud for deep learning (DL) and training [70]. Other ways in which mobile phones using AI-based framework have been helpful during the COVID-19 outbreak include outbreak identification, disease eradication, treatment and infection administration, as well as detection and diagnosis. Fig. 4 shows how the cloud and mobile phones have been used to fight the COVID-19 pandemic.

#### 6) Biosensor Evolution for IoT

After the outbreak of the COVID-19 pandemic, the biosensor became a major device in the fight against the novel coronavirus. At the moment, there are ongoing efforts by many researchers to combine micro-biosensors, microfluidic chips, micro-bioelectronics mini-devices and smartphones into a single device. The objective is to have a portable state-of-the-art clinical diagnostics-based smartphone for real-time point-of-care testing and rapid analysis of clinical and environmental samples in remote and rural areas [74]. For example, with smartphone-based biosensors, many people in rural areas can carry out a simple test at home which could help medical doctors make informed decisions about treatment where medical equipment is not available.

A good example of a biosensor already in use for clinical and diagnostic analysis is the glucose monitor. A similar effort to use biosensor to fight the COVID-19 is the nearly complete development of a single-use wireless biosensor patch 1AX. This patch will be used in early detection and monitoring of the C0VID-19 symptoms including the recording and reporting of respiratory rate, body temperature, and ECG trace in real-time [2], [20]. The rate at which such single-use wireless biosensors will be integrated in H-IoT will largely depend on the cost, simplicity, robustness and reliability of the design [75].

The evolutionary contributions of IoT towards the COVID-19 pandemic fight have been summarized in Table 4.

**TABLE 4:** Summary of Evolution of IoT Technologies During COVID-19

S No.	IoT technology	Description	Evolution	References
1	IoT host hardware	Interconnection of sensors, ubiquitous systems and the internet mounted on host hardware	Drones, robots, chatbots, surveillance, contact tracing, epidemiology, identifying and tracking COVID-19 patients, enforcing quarantine restrictions, in-home patients monitoring, delivery of relief materials to remote residents	[17], [20], [22], [76]
2	AI-based IoT	Instructing systems to recognize, explain and predict a pattern using models based on big data analytics, training, and learning	Risk of infection evaluation, predicting, contact tracing, treatment monitoring, tracking, and identifying COVID-19 outbreak, eradicating fake news, optimize clinical trials of drugs, drones, infodemiology and infosurveillance, biomedicine and pharmacotheraphy	[20], [22], [57], [70], [76]
3	IoT-sourced big data	data collection, analytics, storage and management for real-time decision making	Patients’ health analysis, diagnosis support, and drug/vaccine discovery and production, CT/X-ray imaging manifestation, hematology examination and pathogen detection, and virus spread tracking	[17], [20], [70], [73]
4	Cloud computing	Delivering database, networking, intelligence, and storage over the internet	Outbreak identification, disease eradication, treatment, infection administration, detection and diagnosis, healthcare-specific apps e.g. Saleforce care solution:- helping healthcare providers to manage COVID-19 related requests, Zoom, Google cloud, Azure, Amazon Web helping residents maintain social distancing through virtual reality	[20], [76]
5	IoT biosensors	Sensors converting biological signals into electrical signals	Medical science, outbreak identification, disease eradication, treatment, infection administration, detection, collecting patients’ information, e.g. temperature, ECG trace, respiratory rate, diagnosis, testing and analysis of clinical and environmental samples, e.g. wireless biosensor patch 1AX for detecting and recording COVID-19 cases	[20], [69], [74]

## VII. Challenges of Implementing H-IoT During the COVID-19 Pandemic

Several challenges affect the implementation of healthcare IoT during a pandemic such as the COVID-19. In this section, we present a summary of some of these challenges.

### A. Challenges Associated With IoT Data Collection

Adequate monitoring and control of COVID-19 requires multiple sensor nodes to collect data in various forms for the top-level application to interpret and produce useful information. One of the challenges that would primarily exist in such a scenario is the heterogeneous combination of data collecting devices feeding into the same IoT network. Heterogeneity and vendor specificity in sensor devices leads to a rigid network management system. This becomes an issue when implementing new network policies or deploying applications on an already existing platform [11]. Data collection devices such as wearable location and temperature sensors, thermal imaging devices and smartphones may all be manufactured by different vendors but connected to the same IoT network. Solutions based on software-defined networking have been proposed to increase management flexibility [12], [77] and hence enabling vertical integration of COVID-19 related applications.

Another critical challenge associated with IoT data collection for COVID-19 is the need for data sharing models to support efficiency and ethical data extraction. To aid the smooth integration of data collecting devices in the IoT framework, vendors need some common reference for the configuration of their devices. One suggestion is that a global health data sharing model be developed and adhered to by users, vendors and authorities [5]. There have been reports of ethical guidelines being broken by device data collection as users have shown concern over their health data being used for other than the intended purpose [35], [78].

Security of data collecting devices is another challenge affecting COVID-19 IoT-based management. Snooping into and hacking of sensor devices in the field, have been a concern of H-IoT for a while now and the COVID-19 situation is no exception [79]. Hackers can inject false information in the IoT network potentially misleading healthcare authorities and the general public. The security of these data collection mechanisms is therefore a major concern for stakeholders leading to their reluctance in device and service deployments. Končar *et al.* [80] recommend implementation of secure communication protocols against malware and provision of clearly defined privacy policies to protect users in the IoT framework for COVID-19 management.

In less developed countries, the lack of objects and infrastructure existing as things in IoT is a major data collection challenge. Inadequate resources and governance issues still hinder the rapid development of the required infrastructure for combating COVID-19 [81].

### B. Need for Information Transparency

Providing quality, accurate and appropriate data to stakeholders with the right expertise to make an accurate prediction and take correct decisions during a pandemic for the safety of the community is imperative [17]. For example, during a pandemic people need to have clear information on government decisions, quarantine periods, policies, and travel ban [17]. However, the unorthodox formats and the different sources of data released during a pandemic make the understanding of the reality skewed for the experts to make informed decisions [57]. Most of the data released during the COVID-19 pandemic for example are primarily for public disclosure. Therefore, a huge amount of data is released in news, infographics, texts, map images and table formats making it incongruous and cumbersome for computation.

Also, crowdsourcing and social media are the sources for real-time information during the COVID-19 pandemic. However, the quality, consistency and accuracy of the data collected from these sources remain a challenge due to the lack of standards and guidelines also because almost anyone can upload information to these platforms [57]. Therefore, there is a challenge for the public to be constantly updated with clear and accurate information that will impart awareness about the COVID-19. As a result, only clear information on what the healthcare system knows and does not know about COVID-19 should be given to the citizens so that panic among the general public can be reduced.

### C. Privacy Threats, Cybersecurity Vulnerability, Ethical Controversies and Unanticipated Societal Impacts

The new goldmine of the 21st century is big data. Accordingly, it is anticipated that exerting maximum control over big data will increase countries’ geopolitical standings in the international landscape [5]. Even though the benefits of big data in advancing processes, productivity, and efficiency in different sectors is well documented there are great concerns about trusting others with private data. Other concerns include the nature of data collected, as well as ethical issues with the use and storage of data [76]. For example, China has been criticized for not disclosing the outbreak of C0VID-19 earlier enough as well as its unwillingness in sharing information about its success in containing the pandemic.

Similarly, activists are now advocating for a limit to the amount of personal information that the government can have access to in order not to infringe on people’s privacy. To put this into perspective, despite the attractive benefits from the planned rollout of Huawei’s 5G internet in the US and Europe, the project has been dismissed by respective governments as Beijing’s subtle way of collecting private data under disguise espionage.

### D. Lack of Fundamental Smart City Technology Across Cities to Effectively Fight COVID-19 Pandemic

The integration of IoT technologies as part of the global infrastructure for smart cities still remains crucial to the fight against COVID-19. Currently, most IoT implementations for combating COVID-19 focus on IoT as part of a machine to machine (M2M) framework rather than IoT as part of a global infrastructure. As envisioned in the 5G roadmap, IoT as a global infrastructure is necessary for effective smart city development. Furthermore, 5G infrastructure to support the global IoT framework [66] is still in its infancy stages for most countries. Increased population has been cited as one of the challenges of implementing widespread IoT infrastructure for smart cities. Other challenges hindering smart city development include heterogeneity, cost of operation, information security, system failures and sustainability [18].

The COVID-19 pandemic caught most countries unprepared especially in terms of infrastructure support. In developing countries, smart city governance issues have given rise to delays in infrastructure development. Governments lack the urgency to step up efforts in providing smart infrastructure and accompanying regulatory frameworks for their citizens [81], [82]. While the topic of IoT infrastructure for smart cities has long been in discussion, little progress has been made for most developing countries. Developmental progress has been stuck due to lack of properly laid out guidelines regarding cost, sustainability, waste management, security and regulations. As a result, most countries are yet to benefit from the monitoring and control advantages of COVID-19 that smart city infrastructure promises to provide.

On the other hand, China has the most advanced IoT industry due to its drive to build smart cities [58]. Accordingly, in the fight against the COVID-19 pandemic, the WHO acknowledged that China’s success is largely due to the application of cutting-edge technologies across China’s smart cities. To put this into perspective, IoT is so much entrenched into daily living in China that even basic activity like a bus ride is charged by a facial recognition technology [58]. As a result, China was more technologically prepared to fight the pandemic than most countries.

Conversely, it is unlikely that the concept of smart city technology would be used to monitor and curb COVID-19 in countries where fundamental IoT infrastructures are not available or are still being developed. Therefore, there is a need by governments in different countries to take advantage of the COVID-19 pandemic to imbibe the concept of urban intelligence to enable smart governance e.g. in developing countries. Also, developing countries should start implementing smart city technology in its citizens daily living beyond fighting a pandemic. In 2010, as part of its commitment to building smart cities China for example issued its 12th five-year plan with a blueprint for a social, economic, and political target [58].

### E. Scalability and Dynamic Network Topology Issues, Computational and Energy Limitations

As the number of IoT devices increases there is a need to bring all the devices within a common software framework to ensure a continuous data linkage among the devices [60]. However, there is a huge challenge to increase capacity for storage and integration, as well as standardization, access and use of IoT device data as the number of devices increases [60]. These challenges are further aggravated by limitations of badly structured and integrated healthcare systems, as well as inadequate communication and IoT infrastructures [76].

As a result, there is a need for public health experts, data processing experts and industry stakeholders to offer innovative solutions to healthcare IoT challenges including scalability, security, data integration, interoperability, ethics and privacy [76]. Similarly, limited on-board computational and battery storage capacities hinders devices’ ability for complex on-board data computations and prolong on-going data transmission.

## VIII. Research Gaps and Future Trends

There is a need for more research on the development of automated and rapid alert systems for virus pandemics with expected research advances on COVID-19 and smart applications [23]. We see a growth in IoT based hardware development of thermal sensors and related virus detection tools with a drive towards efficiency and cost reduction. Smartphones are also expected to come with some form of embedded hardware and software designed to aid the combat against a global pandemic such as COVID-19 [83]. Additional hardware in terms of wearable devices for detection such as the smart helmet [26] and also for track/trace requirements are expected in a more advantageous and cost-effective form.

Data collection of COVID-19 related parameters through an IoT framework would require a connection of multiple heterogeneous devices. To maximize network management efficiency through the support of multiple vendor devices and applications there is a need for implementation of software-defined IoT. SDN-based IoT would enable the existence of a logically centralized controller to monitor and collect data from multiple devices spread over large areas, while allowing for scalability and flexible management [11], [12]. Another advantage is the support for vertical integration of COVID-19 related applications that could easily be interchanged as implementation policies vary. Applications in SDN-based IoT frameworks have been envisioned to exist as replaceable and interchangeable modules [84] that would support rapidly changing policies and governance as in the case of COVID-19.

Big data analytics also provide a promising driving force behind IoT for combating global virus pandemics [85], [86]. Several IoT technologies backed by big data and AI are being proposed but with the realization that they might not reach their full potential at this particular period. The major drawback being the unwillingness of communities to share personal data such as location and other relevant health data [5]. We however envision a future with a general acceptance towards data sharing for the benefit of a global community. As part of an evolved IoT paradigm in an era of rapidly spreading virus pandemics, governments and society are expected to appreciate how technology can limit the virus spread and save lives. Further research and developments are expected towards improved data security and privacy for IoT architectures in a bid to improve confidence levels for data sharing among communities. There is also a need for the development of data sharing models to support this data-centric approach.

In terms of prospects for IoT policy making, there is a need for proficiency in domain knowledge. During a pandemic, IoT experts are the professionals using extensive engineering, scientific and computational skills to collect and analyze data daily at the frontline. However, most of these experts are only skilled in computer science and statistics but are not proficient in domain knowledge. Domain expertise is crucial in isolating actionable insights, ratifying important forecasts, making appropriate decisions and appraising potential impact. Therefore, there is a need for synergy between data science and domain knowledge in the feasibility of deploying and measuring the improvement of IoT actions. As well as, the anticipated and unanticipated social, economic, and political consequences of taking such actions [57].

Governments and international health organizations like the WHO should create more policies that will engender coordinated efforts among stakeholders. Such as encouraging inter-sectoral collaboration, and increased resources e.g. more masks, PPEs, medical supplies for the fight against the pandemic [87]. A perfect example of collaborative efforts that ought to be encouraged, is the instance of scientists from Doherty Institute in Australia. They were able to grow a virus similar to the COVID-19 in their Virus Identification Laboratory from data collected from Chinese scientists through the Global Initiative on Sharing All Influenza Data (GISAID) [5].

Generally, public health systems will benefit from the fast-evolving mobile phone penetration, advancements in cloud computing, and the development of health-specific mobile applications. The capacity of IoT in analyzing health information search, public communication and behavior over the internet will improve by advancements in the fields of infodemiology and infosurveillance [76]. Similarly, the security of healthcare IoT will be bolstered by advances in cryptographic technologies, e.g. blockchain [10].

## IX. Conclusion

As the world undergoes an ongoing battle against the deadly COVID-19 virus, we see rapid adoptions of IoT technologies towards monitoring and reduction of the virus spread. This article reviewed several IoT technologies being adopted in combating COVID-19. The nature of IoT adoption for pandemic management was illustrated and expressed in the form of an ecosystem consisting of hardware, software, data analytics and ethical conditions. It was realized from the review that fighting a global pandemic relies heavily on data collection which ultimately results in big data. This was highlighted as a major challenge citing privacy and security concerns in allowing IoT devices to collect personal data required for smart pandemic management.

COVID-19 has posed an IoT technological challenge which has resulted in the development of new forms of sensor deployments and IoT techniques. Therefore, as part of the evolution analysis and review, this article looked at the state of healthcare IoT before and now during the existence of COVID-19. Generally, a major finding of this research article has been the realization that for IoT to effectively aid the fight against a global pandemic, there is a need for social acceptance and security in data sharing for IoT management systems. Increased availability of quality accurate data will improve the control and monitoring of a fast-spreading virus such as COVID-19.
